# Low-Dose Prednisolone for the Prevention of Recurrent Relapses in Nephrotic Syndrome Triggered by Regular Hospital Visits: A Case Report

**DOI:** 10.7759/cureus.81247

**Published:** 2025-03-26

**Authors:** Hiroaki Kanai, Miwa Goto, Anna Kobayashi, Emi Sawanobori

**Affiliations:** 1 Department of Pediatrics, Suwa Central Hospital, Chino, JPN; 2 Department of Pediatrics, Faculty of Medicine, University of Yamanashi, Chuo, JPN

**Keywords:** nephrotic syndrome, prednisolone, psychological stress, regular hospital visits, relapse

## Abstract

Hospital visits causing psychological stress can trigger nephrotic syndrome relapse in children. While there are reports on preventing relapse during respiratory infections by using low-dose steroids, no reports exist on preventing relapse from other triggers. We describe a case of a patient with repeated relapses triggered by regular hospital visits, which were successfully prevented by administering low-dose prednisolone before the visits. A 14-year-old boy with steroid-dependent nephrotic syndrome was referred and started on mycophenolate mofetil. During the following 14 months, there were nine regular hospital visits. Up to the third of four relapses, urinary proteins appeared on the day of the hospital visit and five and three days before the hospital visit. He experienced two instances of transient proteinuria, with positive urine protein test results on the day of a regular visit. Regular hospital visits were judged to trigger a relapse. At age 16, he was started on prednisolone at 20 mg (approximately 15 mg/m² or 0.5 mg/kg) for prophylaxis five days before regular hospital visits. Thereafter, he no longer experienced relapse or transient proteinuria. However, at age 19, he experienced a relapse related to a hospital visit despite prophylaxis with prednisolone. Since regular hospital visits were discontinued and he was placed under the regular care of a local doctor, prophylactic administration of prednisolone before hospital visits was discontinued. Thereafter, the mycophenolate mofetil dose was tapered off when he was 20 years old. He did not experience a relapse again until age 23. The case shows that low-dose prednisolone administration can prevent hospital visit-related relapse as well as relapse during respiratory infections.

## Introduction

Nephrotic syndrome (NS) is the most common manifestation of glomerular disease in childhood. It is characterized by massive proteinuria, hypoalbuminemia, and/or edema. Most patients with pediatric idiopathic NS respond to steroid treatment; however, approximately 30%-40% of children develop frequent-relapsing or steroid-dependent NS. Frequent relapse is defined as two or more relapses within six months of an initial response or four or more relapses in any 12-month period. Steroid dependence is defined as two consecutive relapses during steroid therapy or within two weeks of its cessation. NS relapse can be triggered by immunological stimuli, such as infections, vaccinations, and allergic episodes [1]. Situations that cause psychological stress, such as school events, domestic events, and hospital visits, can also act as triggers [2].

Upper respiratory tract infection (URTI) is the most common trigger of relapse, and some prospective studies have indicated that relapses are significantly reduced when the maintenance dose of prednisolone (PSL) is increased or when a low daily dose of PSL is administered during URTI [3,4]. However, there are no reports on the prevention of relapses related to other triggers. Herein, we describe a case of repeated relapses triggered by regular hospital visits, which were prevented by administering a low dose of PSL before the visits.

## Case presentation

**Figure 1:**
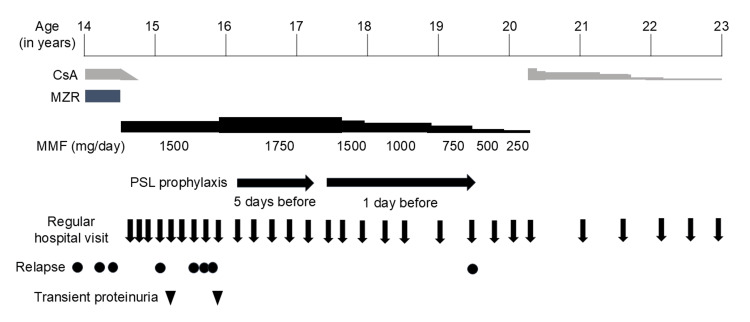
Patient’s clinical course CsA: cyclosporine A; MZR: mizoribine; MMF: mycophenolate mofetil; PSL: prednisolone

A male patient with steroid-dependent NS who developed NS at four years of age and had 36 previous relapses was referred to our hospital for further treatment at 14 years of age. He had been taking mizoribine (MZR), cyclophosphamide, and azathioprine at different times but experienced frequent relapses and was steroid-dependent. Therefore, he was administered cyclosporine A (CsA) at 11 years of age and was in remission the following year. However, he experienced four relapses during the following year and was started on MZR at 14 years of age. Nevertheless, he experienced three relapses in the following eight months (Figure 1).

Upon referral to our hospital, his height was 149.7 cm (-2.61 standard deviations) and weight was 44.7 kg (BMI 19.9), indicating short stature but not obesity. No hypertension was observed (94/65 mmHg), and the physical examination findings were unremarkable. After his initial visit to our hospital, he started taking mycophenolate mofetil (MMF) at a dose of 1,500 mg/day; MZR was discontinued, and the CsA dose was tapered off. Fourteen months after starting regular visits to our hospital, he relapsed four times and was treated with PSL. During this period, he had nine regular hospital visits. During the first three of the four relapses, urinary proteins in the first morning urine were detected either on the day of the hospital visit or five and three days prior to the regular hospital visit (corresponding to the fourth, seventh, and eighth visits, respectively). On the first occasion, a dipstick test revealed 2+ for protein, and the urinary protein-creatinine ratio (uP/Cr) was 0.34 g/gCr. Proteinuria persisted for four consecutive days, increasing to a uP/Cr of 0.58 g/gCr, leading to a diagnosis of relapse [5]. On the second occasion, the dipstick test revealed 4+ for protein, and the uP/Cr was 1.65 g/gCr at the time of the hospital visit. Proteinuria persisted for an additional seven consecutive days, increasing to a uP/Cr of 3.42 g/gCr. On the third occasion, the dipstick test revealed 4+ for protein, and the uP/Cr was 1.01 g/gCr at the time of the hospital visit. Proteinuria persisted for another five consecutive days, increasing to a uP/Cr of 2.61 g/gCr. Additionally, transient proteinuria was observed during a fifth hospital visit, when the patient tested positive for urinary proteins at 1+ by dipstick testing and the uP/Cr was 0.24 g/gCr; this resolved spontaneously within a week. During the third relapse, we suspected that the patient’s regular hospital visits were the relapse trigger. In the fourth relapse, proteinuria at the time of the hospital visit, measured 4+ by dipstick testing with a uP/Cr of 2.61 g/gCr, appeared more than two weeks before the regular visit. However, the patient also experienced transient proteinuria during a ninth hospital visit, measured 1+ by dipstick testing with a uP/Cr of 0.55 g/gCr, which resolved spontaneously within a week, similar to the fifth hospital visit. No signs of infection were noted at the time of these occurrences of proteinuria, and no significant lifestyle changes were observed.

These findings indicated that the regular hospital visits were triggers for both relapses and transient proteinuria. Moreover, two of the three relapses associated with hospital visits occurred when the patient was not taking PSL, with one occurring while the patient was on a low dose of 5 mg every other day. On two occasions of transient proteinuria, the patient received higher doses of PSL (15 and 20 mg every other day). Of the four instances when proteinuria was not observed, only once was PSL not taken. Therefore, we considered that steroids prior to the regular hospital visits could be effective in preventing relapse. Subsequently, when he was 16 years old, he was started on daily PSL at a dose of 20 mg (equal to 15 mg/m^2^ or approximately 0.5 mg/kg) for prophylaxis five days before regular hospital visits.

Thereafter, he regularly visited the hospital approximately every three months and experienced no relapse or transient proteinuria. At a regular visit at 17 years of age (his sixth visit after starting prophylactic administration of PSL), he had forgotten to take PSL five days prior and only began taking it the day before the visit. However, no urinary protein was detected. Hence, at subsequent regular visits, he took PSL from the day before the visit, and proteinuria was not observed. Starting at age 17, MMF was tapered. At 19 years of age, he experienced a relapse related to a regular hospital visit despite PSL administration. Since regular visits to our hospital were discontinued after that visit and he was placed under the regular care of a local doctor, prophylactic administration of PSL prior to regular hospital visits was discontinued accordingly. Thereafter, he visited the local doctor approximately every six months, and MMF was fully tapered off by age 20. However, because his atopic dermatitis worsened, the dermatologist prescribed CsA at a dose of 150 mg/day at 20 years of age. Thereafter, the dose was gradually tapered off at 22 years of age. The patient did not relapse until 23 years of age.

## Discussion

In pediatric NS, psychologically stressful episodes, such as school events, domestic events, and hospital visits, can trigger relapse; moreover, these have also been reported to be associated with the appearance of proteinuria in children with NS [2,6]. However, in clinical practice, establishing a causal relationship between relapse and psychological stress is often difficult. We initially suspected that the patient’s regular hospital visits triggered a third relapse (eighth visit) after starting regular visits to our hospital. Transient proteinuria was observed at the subsequent hospital visit (ninth visit). When we retrospectively examined the results of urinary proteins from past visits, we found that proteinuria had also been observed at the fourth, fifth, and seventh visits, with relapses occurring at the fourth and seventh visits. Therefore, we concluded that regular hospital visits triggered his relapse. Since the patient had been recording his urine test results at home daily, we could identify when proteinuria first appeared and establish a causal relationship. Relapses without apparent triggers, such as the common cold, often occur within three days of a regular hospital visit [2]. Therefore, in pediatric NS with frequent relapses, clinicians should instruct patients to perform daily urine tests for at least one week prior to the regular hospital visit.

The mechanism of relapse due to psychological stress in NS is unknown; however, endogenous cortisol release leading to dysregulated immune responses has been suggested [6]. Other relapsing diseases, such as inflammatory bowel disease, bronchial asthma, multiple sclerosis, and systemic lupus erythematosus, are also associated with psychological stress [7-10]. The pathophysiology of psychological stress in each disease is unclear, but studies on bronchial asthma have shown that psychological stress leads to the activation of the hypothalamic-pituitary-adrenal (HPA) pathway [11]. This is followed by the secretion of cortisol, which enhances T-helper 2 (Th2) and T-helper 17 (Th17) immune responses and attenuates regulatory T (Treg) cell responses, which are closely linked with exacerbation due to allergic airway inflammation. Moreover, exogenous steroids used in the treatment of acute exacerbations are effective because they are administered in higher doses than the increase in endogenous cortisol caused by psychological stress, metabolized more slowly, and remain in the body for a longer period, although the extent to which psychological stress increases endogenous cortisol remains unknown [12]. Although the exact immunological mechanism underlying NS relapse is unknown, several studies have focused on T- or B-cells, particularly on T-cell-mediated immunological mechanisms [13]. A T-helper 1/Th2 imbalance in favor of the Th2 compartment, an increased Th17/Treg ratio, an upregulation of Th17 responses, and a downregulation of Treg responses have been recognized [13,14]. This mechanism is similar to that of acute exacerbation of bronchial asthma. In addition, the mechanism by which increasing PSL during URTI prevents relapse is thought to involve steroids suppressing the release of cytokines from T-cells activated by the infection [4]. Therefore, in the present case, it is speculated that the HPA pathway was activated by the psychological stress associated with regular hospital visits, leading to the release of endogenous cortisol, which activated T-cells and dysregulated the immune response, resulting in relapse or proteinuria. Thus, the dose of PSL (15 mg/m^2^, approximately 0.5 mg/kg) administered in this case was sufficient to suppress T-cell activation. The patient’s clinical course, with no proteinuria observed for over three years after starting PSL prophylaxis, supports the hypothesis regarding this mechanism.

Two aspects of this case remain unclear. First, while PSL is typically administered following the onset of infection-related symptoms such as a runny nose and sore throat, to prevent relapses triggered by respiratory tract infections, the appropriate timing for initiating prophylactic administration of PSL to prevent hospital visit-related relapse remains uncertain [3,4]. Initially, we started the prophylactic administration of PSL five days before the patient’s regular hospital visits, based on the timing of previous relapses related to these visits. Following this, we adjusted the starting date of PSL administration to the day before the hospital visit, and the patient experienced no relapses for over two years. However, at 19 years of age, he experienced a relapse related to a regular hospital visit despite PSL administration. For reasons unknown, the administration of PSL may have had a partial placebo effect. Second, it is unclear why the patient did not experience any relapses even without prophylactic administration of PSL, after transitioning from regular visits to our hospital to local hospital visits. Although CsA was initiated after MMF was tapered off, it was gradually reduced and tapered off at 22 years of age. Given that the patient had experienced relapses even while taking high-dose MMF, it is unlikely that CsA alone was responsible for preventing relapses. Other possible explanations include a natural reduction in the disease activity of NS as the patient matured or reduced psychological stress from visiting a local hospital, which was closer and more convenient [15]. However, the exact reason remains unclear.

## Conclusions

Clinicians should be aware that regular hospital visits, which lead to psychological stress, can trigger relapse in patients with pediatric NS. Prophylaxis with a low dose of PSL should be considered to prevent relapse if the triggers are known and predictable in similar cases.
